# Looking for immortality: Review of phytotherapy for stem cell senescence

**DOI:** 10.22038/IJBMS.2019.40223.9522

**Published:** 2020-02

**Authors:** Hourieh Tousian, Bibi Marjan Razavi, Hossein Hosseinzadeh

**Affiliations:** 1 Department of Pharmacodynamics and Toxicology, School of Pharmacy, Mashhad University of Medical Sciences, Mashhad, Iran; 2 Targeted Drug Delivery Research Center, Pharmaceutical Technology Institute, Mashhad University of Medical Sciences, Mashhad, Iran; 3 Pharmaceutical Research Center, Pharmaceutical Technology Institute, Mashhad University of Medical Sciences, Mashhad, Iran

**Keywords:** Cellular senescence, Endothelial progenitor cells, Inflammation, Panax ginseng, Regeneration stem cell, Resveratrol, Telomerase

## Abstract

In this paper, we discussed natural agents with protective effects against stem cell senescence. Different complications have been observed due to stem cell senescence and the most important of them is “Aging”. Senescent cells have not normal function and their secretary inflammatory factors induce chronic inflammation in body which causes different pathologies. Stem cell senescence also has been investigated in different diseases or as drug adverse effects. We searched databases such as Embase, Pubmed and Web of Science with keywords “stem cell”, “progenitor cell”, “satellite”, “senescence” and excluded keywords “cancer”, “tumor”, “malignancy” and “carcinoma” without time limitation until May 2019. Among them we chose 52 articles that have investigated protective effects of natural agents (extracts or molecules) against cellular senescence in different kind of adult stem cells. Most of these studies were in endothelial progenitor cells, hematopoietic stem cells, mesenchymal stem cells, adipose-derived stem cells and few were about other kinds of stem cells. Most studied agents were resveratrol and ginseng which are also commercially available as supplement. Most protective molecular targets were telomerase and anti-oxidant enzymes to preserve genome integrity and reduce senescence-inducing signals. Due to the safe and long history of herbal usage in clinic, phytotherapy can be used for preventing stem cell senescence and their related complication. Resveratrol and ginseng can be the first choice for this aim due to their protective mechanisms in various kinds of stem cells and their long term clinical usage.

## Introduction

Although stem cells with high capacity of regeneration and self-renewal have been distributed in whole body, human isn’t immortal. As age increases, man experiences aging phenomenon and its complications such as cardiovascular diseases, immunodeficiency and brain dementia (1). Aging is due to insufficient stem cells regeneration (2). Decrease in the number or function of stem cells happens by aging. There are several mechanisms induce intrinsic stem cells aging such as telomere shortening, accumulation of DNA damages, negative epigenetic alteration and exhaustion of proliferative potential. Cellular senescence is arrest in cell cycle and lack of cell cycle progression causes loss of stem or progenitor cells which are necessary for tissue repair, regeneration and normal cell turn over (3-5). Cell cycle has different checkpoints and mechanisms to preserve DNA integrity and to repair its damages. If DNA repair systems fail, apoptotic signals will begin and cells will become apoptosis to eliminate cells with damaged genome. If signals are not enough to start apoptosis, cells will undergo a persistent proliferative arrest known as cellular senescence. Apoptosis or senescence due to genomic damage is protective barrier against cancer. Whenever these barriers fail, cancerous cell division will begin (6-11). P53, p16, p19 (p14 in human) and retinoblastoma protein (Rb) are tumor suppressor proteins that work in this cell cycle checkpoints. They regulate cellular proliferation negatively, to promote DNA repair; As genomic damages increase by aging, their expression will increase (12). Senescent cells secrete pro-inflammatory factors which create senescence-associated secretory phenotype (SASP). SASP attract immune cells to clear senescent cells. The accumulation of many senescent cells due to high rate of formation or low rate of clearance, led to chronic inflammation in body that results in different pathologies. Also the senescence of stem cells has been investigated in different non-aging pathologies such as obesity, diabetes, hyperlipidemia and drug adverse effects (13-16).

There are 240 studies about natural products with anti-aging effects until May 2017 (17). In this paper, we reviewed some clinical usage of stem cells, related problem to their senescence (18) and all studies about natural agents that prevent stem cell senescence with their protective mechanisms. 

## Materials and Methods

We searched databases such as Embase, Pubmed and Web of Science with keywords “stem cells”, “progenitor cell”, “satellite”, “senescence” and excluded keywords “cancer”, “tumor”, “malignancy” and “carcinoma” without time limitation until May 2019. Among 2014 results, we chose 52 articles that investigated protective effects of natural agents (extracts or molecules) against cellular senescence in different kind of stem cells.

***Protective effects of natural agents against senescence of different kind of adult stem cells***

**Figure 1:**
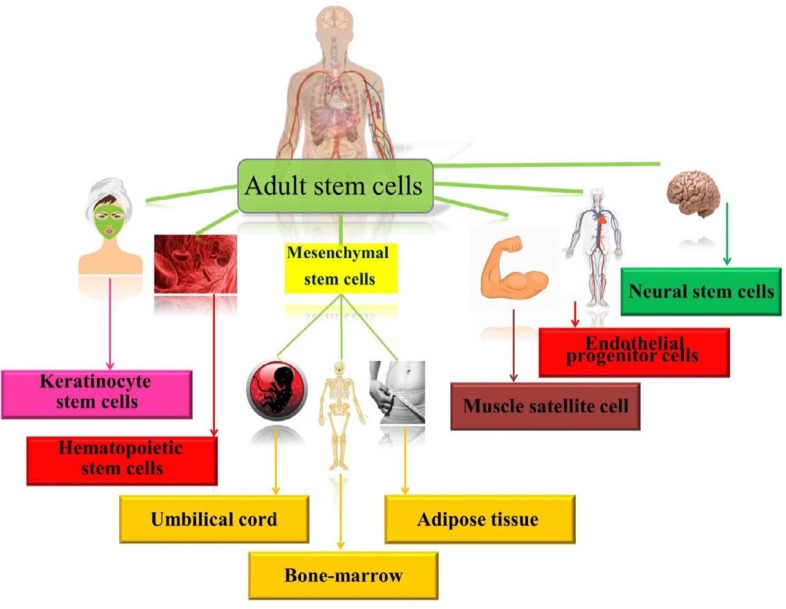
The schematic of stem cell sources

There are different kinds of adult stem cells in different organs. Most studied stem cells are endothelial progenitor cells (EPCs), hematopoietic stem cells (HSCs), mesenchymal stem cells (MSCs). Keratinocyte stem cells (KSCs), neural stem cells (NSCs), cardiac progenitor cells (CPCs) and myeloblasts are other kinds of adult stem cells that their senescence have been studied (Figure 1). The protective mechanisms of natural agents in senescence of stem cells are summarized in Table 1.

***Endothelial progenitor cells (EPCs)***

EPCs play important roles in homeostasis of the vasculature and formation of new blood vessels (19). Decrease in their number and functions are associated with aging and atherosclerotic processes (20, 21). EPCs from individuals at high risk of atherosclerosis become senescent more rapidly than individuals at low risk of atherosclerosis (22). Senescent EPCs show limited migratory and proliferative capacity for cell therapy in ischemic diseases (20). Obese patients exhibit premature EPCs senescence and decline in their numbers (23). Angiotensin II (Ang2), which has increased in hypertension disease, accelerates EPCs senescence and reduces their differentiation capacity (24). 

*In vitro studies*

The increasement of Ang2 plays an important role in high blood pressure (BP) and inhibition of its receptor, AT_1_R, or its activator enzyme, angiotensin converting enzyme, will decrease BP (25). In hypertension disease because of senescent EPC endothelial repair capacity is reduced , so, endothelial function is impaired (26). 

Ang2 diminished telomerase activity and HO-1 level in EPCs. 1.0, 2.0 and 5.0 μM of oleuropein (main bioactive phenolic constituent of *Olea europaeal* leaves) have shown hypotensive effects and oleacein (predominant phenolic constituent of olive oil extra virgin) prevented senescence induced by Ang2 in human-EPCs (h-EPCs) by decreasing ROS production, elevating telomerase activity and mRNA expression of transcription factor Nrf_2_ and heme oxygenase-1 (HO-1). Nrf2 controls basal and inducible expression of anti-oxidant genes such as HO-1 in the cell (27). HO-1 has an anti-inflammatory role in EPCs. 

In addition, these agents improved re-endothelialization ability of injured arterial wall and neovascularization of ischemic tissue (28). It’s well known that Mediterranean diet with olive oil showed protective effect in cardiovascular system (28).

Similar to oleuropein and oleacein, *Aronia melanocarpa* extract (1-25 μg/ml), which is rich in anthocyanins, decreased cellular senescence induced by Ag2 in h-EPCs. This extract elevated telomerase and Nrf2 activity, HO-1 expression and reduced intracellular ROS production (29). This agent can be considered for EPCs protection in hypertension disease.

Ginsenoside Rg1, that is a class of steroid glycosides and triterpene saponins, has been found exclusively in the plant genus Panax (ginseng). A study showed that 5 μM of ginsenoside Rg1 increased telomerase activity, so, prevented telomere shortening and senescence in serial transplantation of h-EPCs (30). In another study, 200 μg/ml of sun ginseng (which is processed at 120 ^°^C to form different Rg subclasses) prevented senescence in h-EPCs and enhanced their repairing mechanisms. The mechanisms of its anti-senescence effects have not been studied (31). 

*Ginkgo biloba* extract (25 mg/l) inhibited senescence of h-EPCs in prolonged cultivation. Its protective mechanism was telomerase activity induction via PI3K/AKT pathway (32). 

Moreover, 1.0 mM of puerarin (a major effective ingredient extracted from the traditional Chinese medicine Ge-gen (*Pueraria lobata*) increased telomerase activity through increasing Akt phosphorylation and inhibited senescence in h-EPCs (33). 

A natural polyphenolic compound in some plants such as grape (34, 35), resveratrol (0.110 μM), increased hTERT, PPARγ/HO1 protein expressions, resulted in suppression of oxidative stress and prevented senescence in h-EPCs. PPARγ similar to Nrf2 transcriptionally regulates the expression of HO-1. HO-1 inhibits the activity of NADPH oxidase so decreases the level of intracellular ROS (36). Furthermore, resveratrol (10, 25 or 50 mM) increased telomerase activity through increasing phosphorylation of Akt and prevented senescence in h-EPCs. Also these studies revealed that higher concentrations of resveratrol induced senescence by itself (37). In another study, 1, 10 and 50 µM of resveratrol increased the phosphorylation and activation of Akt through PI3-K/Akt pathway, so, increased hTERT expression and telomerase activity and decreased senescence in h-EPCs (38). The prevention of telomere shortening is a protective mechanism against cellular senescence. The Akt activation also inhibits transcriptional activity of fork head transcription factor (FOXO3a). FOXO3a increases anti-oxidant capacity of the cell. Down-regulation of FOXO3a results in increasing level of ROS and genomic damage. This damage leads to activation of P53/P21 pathway and cellular senescence (39). In MSCs high concentrations of resveratrol induced senescence via activating β-catenin and change of Sirt-1 level (40-42). 

Oxidized low-density lipoprotein (ox-LDL) inactivated telomerase and induced EPC senescence, which led to impairment of their proliferative capacity and network formation (43). 

Polysaccharides of Angelica sinensis (100μg/ml or 20μg/ml) inhibited ox-LDL-induced senescence in rat-BM-generated EPCs (r-EPCs) by elevating telomerase activity via NADPH oxidase and Akt/hTERT pathways. Oxidative stress regulates telomerase activity. These polysaccharides inhibited NADPH oxidase activity and decreased ROS, so, restored telomerase activity by two mechanisms (44). 

Pre-treatment by 0.7 mg/ml of *Triticum sativum* grain powder increased glutathione peroxidase (GPx-1), superoxide dismutase 2 (SOD2), Nrf-2 translocation into the nucleus, HO-1 expressions and 0.35 mg/ml of bean lysate increased GPx-1 and SOD2 expressions. Both of them decreased ROS generation and attenuated senescence of h-EPCs exposed to H_2_O_2_. In addition different studies showed Nrf_2_ translocation into the nucleus activates anti-oxidant genes such as catalase, GPx-1 and SOD2 (45). 

Studies have indicated that high glucose induces EPCs senescence via p38 mitogen-activated protein kinase (MAPK) pathway and reduces their proliferative, migratory and tube formation capacity (46, 47). MAPK is a mediator of inflammation and stress responses, involves in the control of cell cycle and cellular proliferation (39). Pathological ROS production induces MAPK and p38 activation, contributes to p53-induced replicative senescence (48). So, if anti-oxidant capacity of the cell is increased by different mechanisms such as HO-1 protein expression, ROS and its related post signals such as MAPK will be abolished.

 Red Yeast Rice (50 *μ*g/ml) inhibited oxidative stress and senescence induced by high-glucose in h-EPCs through Nrf_2_ nuclear translocation and HO-1 protein expression (49). 

In another study, 12.5–50 mg/ml of silymarin (a flavonolignan complex isolated from *Silybum marianum*) increased telomerase activity and protected h-EPCs against senescence induced by rapamycin (0.1 ng/ml). Rapamycin inhibits mTOR and accelerates EPCs senescence and may impairs reconstruction of injured atrial (50). inhibition of mTOR decreases telomerase activity in cancer stem cells (51). The exact protective mechanism of silymarin needs further investigation

In another study, 0.1, 1, 10, and 30 μg/ml of fucoidan (marine sulfated polysaccharide extracted mainly from various species of brown algae and brown seaweed) decreased p21 and increased anti-senescence protein, SMP30 (senescence marker protein 30), in human endothelial colony-forming cells (h-EFCF) and decreased senescence through Akt and extracellular signal-regulated kinase (ERK) phosphorylation in h-EPCs in dose dependently manner. Akt phosphorylation decreases p21 and increases SMP30 protein level. Phosphorylated ERK decreases p21 and promotes cell cycle in EPCs. SMP30, as a marker of senescence and aging, protects cell against oxidative stress (52). In response to oncogenic threat, ERK as a tumor suppressor phosphorylates multiple targets and arrests the cell cycle (53). Oxidative stress decreases SMP30 expression and life span of the cell (54). SMP30 participates in plasma membrane Ca^2+^ pumping activity and vitamin C biosynthesis which both regulate insulin secretion in pancreatic beta-cells of mice (55). 

*In vivo studies*

Curcumin, 1000 mg/kg/day, (a bright-yellow chemical isolated from the roots of *Curcuma longa *(56) for 14 days reversed senescence induced by glucose in EPCs of type-1 diabetic mice. It also overexpressed vascular endothelial growth factor and angiopoetin-1 in EPCs and improved neovascularization (57). This effect is considerable in metabolic syndrome disease. EPCs protection in diabetic patient is important aim to reduce their cardiovascular complications.

*Human studies*

In healthy volunteers, intake of 250 ml/day red wine for 3 weeks, reduced glucose-induced senescence in EPCs through enhancement of mRNA and protein expressions of PI3K/Akt/eNOS pathway (58). Intake of 100 ml/day red wine for 3 weeks or incubation EPCs from healthy, young subjects with 1% red wine or 50 μM resveratrol decreased cellular senescence induced by TNF-α, due to elevated NO by eNOS (59). NO bioavailability or eNOS activity increasement can elevate h-TERT and telomerase activity (60). In addition, red wine contains various anti-oxidant polyphenol derivatives of grape such as resveratrol. The cardiovascular protective mechanisms of resveratrol on isolated tissues or organs have been well-described (61). Although there is evidence for beneficial cardiovascular effect of mild to moderate red wine consumption, there is no clinical advice to initiate daily wine consumption in the literature (62). Moreover, because of alcohol content, long-term resveratrol-enriched wine products consumption causes hepatic adverse effects. So, other food products and nonalcoholic beverages could be considered as alternative resveratrol-enriched wine products (63).

**Figure 2:**
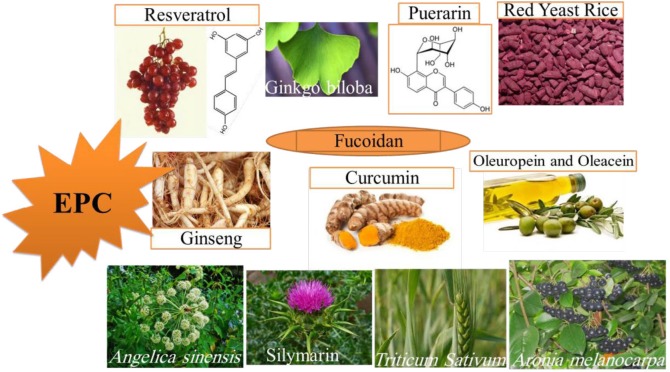
Natural agents that have shown protection against endothelial progenitor cell (EPC) senescence

There are different reasons for EPCs senescence. Moreover, there are various agents for EPCs protection against senescence. These agents could be prescribed for elderly population to improve their EPCs efficacy and to reduce age-related cardiovascular complications. We should also, consider time and dose-dependent effects of therapeutic agents in short and long term consumption on cellular senescence as mentioned for resveratrol and fucoidan. Maybe one agent shows desirable effects in short-term or low-dose but shows adverse effects in long-term or high-dose usage (Figure 2). 

***Hematopoietic stem cells (HSCs)***

HSCs play critical roles in blood coagulation, oxygen transportation and immune system, so that, HSC senescence causes blood dysfunction (64). The infusion of HSCs has been shown positive results in different conditions, for example infusion in renal transplantation decreased rejection episodes and immunosuppressive drug requirements (65). The infusion of HSCs after chemotherapy in rheumatoid arthritis drug resistance patients, modified their disease (66), and in diabetic patients improved mean fasting blood sugar, post prandial blood sugar, HbA1c and decreased glutamic acid decarboxylase antibodies (67). Ox-LDL induced HSC senescence by oxidative stress and reduced telomerase activity (68). Transplanted HSCs due to rapid telomere shortening undergo accelerated senescence. Senescent HSCs have shown a reduction in clonal stability, homing, engraftment and biased lineage commitment (64). 

*In vitro studies*

Tianshengyuan-1(Chinese herbal medicine, a liquid extraction of multiple Chinese herbs) at 31.2 μg/ml and 62.5 μg/ml concentrations increased telomerase activity by enhanced expression of TERT gene so decreased senescence in umbilical cord blood-HSCs (69). 

Ten μM of ginsenoside Rg1 prevented senescence induced by tert-butyl hydroperoxide (t-BHP) in HSCs via reduction of P16 gene expression and p19-p53-p21 signaling pathway. Rg 1 down-regulated p16, cyclin D1 and p21 and up-regulated CDK4, cyclinE and CDK2. P16-Rb and p19-MDM2-p53-p21 signaling pathways are involved in cellular senescence. P16 can inhibit, CDK4/6 binding to cyclinD1 so blocks Rb phosphorilation and arrests cell cycle. P19 binds to MDM2 and prevents the degradation of p53. P53 induces p21 and p21 inhibits the CDK4/6-cyclinD1 complex-induced Rb phosphorylation and activity of the CDK2/cyclinE complex and arrests cell cycle which results in cellular senescence (70). In another study, 10 μM Rg1 via Sirt6 induction, down-regulated NF-κB and exhibited anti-senescence effect in HSCs of mice (71). Also, 10 μM of Rg1 decreased p16 mRNA and protein level and delayed senescence in HSCs (72). 

*In vivo studies*

Pretreatment with Ginsenoside Rg1 better than treatment, increased expression of Sirt1 and decreased NF-κB. Sirt1/NF-κB signal axis prevented senescence in HSCs of D-galactose aging model rats (73). Ten μM of ginsenoside Rg1 decreased P16 and Rb protein, increased the expression of CDK2, CDK4 and cyclin E (cell cycle proteins), so, inhibited the senescence of HSCs *in vitro *and in mice. Senescenceassociated p16Rb signaling pathway was alleviated in mice by elevation of telomerase activity and restoration of telomere length (74). Ionizing radiation or chemotherapy approaches in cancer patients increased senescence of HSCs and impair their self-renewal ability (75) and induced long-term bone marrow suppression through NADPH oxidase 4 (NOX4)-derived ROS (76). HSCs/HPCs from irritated mice, treated or pretreated by 20 mg/kg of Rg1, showed less senescence during serial transplantation. Rg1 reduced cyclin D1, p16-Rb and p19-p53-p21 signaling pathway and up-regulated the expression of CDK4, CDK2 and Cyclin E proteins (77). Twenty mg/kg/day of Rg1 for 7 days before radiation, increased SOD activity and decreased HSCs/HPCs senescence in mice (77). Treatment by 20 mg/kg/day of ginsenoside Rg1 7 days after irradiation in mice, exposed to 6.5 Gy X-ray, increased SOD activity result in decrement of DNA damage and P16 and P21 expressions in mice thus ginsenoside Rg1 decreased senescence in HSCs/HPCs (78). 

Fifty mg/kg treatment with theaflavin (a polyphenolic compound from black tea) one day before and up to 7 days after irradiation reduced the ROS level, p16 and senescence of HSCs in irradiated mice by up-regulating of Nrf-2 level (79). 

Mice feeding with 3 g/day of *Siraitia grosuenori* showed less senescent HSC due to ROS level decrement and down-regulation of p21, p53 and p16 proteins (80). 

Pretreatment or treatment with 20 mg/kg of resveratrol after total body irradiation reduced HSC senescence. Resveratrol by NOX4 and Sirt1 increased expression of SOD1 and GPX1 so inhibited ROS production. This agent alleviated long term bone marrow injury (76). 

Different proportions of astragalus-angelica (10:1, 5:1, 1:1 and 1:5) or 6 g/kg astragalus or 3 g/kg angelica inhibited senescence of BM-HPCs in mice with BM suppression due to cyclophosphamide (an anti-cancer drug belongs to alkylating agents class) administration (81).

Mice treated by 200 mg/kg of *A. sinensis* polysaccharides during X-ray radiation showed less HSC senescence due to telomerase activity increasement and p53 down-regulation (68). *A. sinensis* (200mg/kg) polysaccharide in D-galactose induced aging mice, increased antioxidants capacity, decreased DNA damages, P16-RB, P19-P21 and excessive activation of Wnt/beta-catenin signaling, so, prevented senescence in BM-HSCs/HPCs (82). The excessive activation of Wnt/*β*-catenin signaling causes stem cell senescence (82). C-MYC and cyclin D1 have been reported as targets of the wnt/β-catenin pathway (83, 84). 

**Figure 3:**
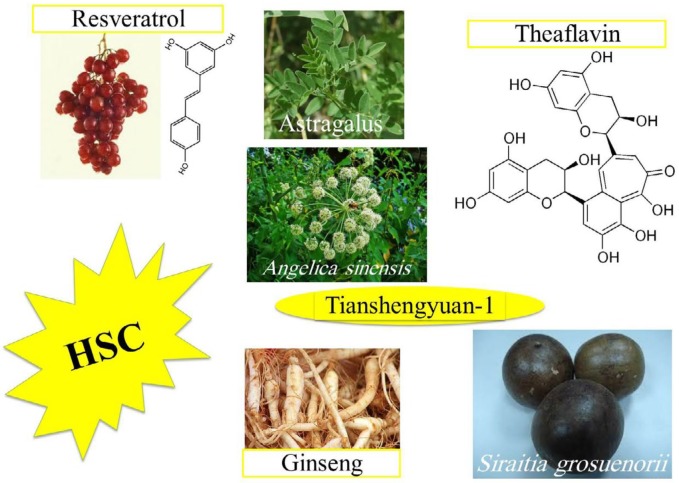
Natural agents that have shown protection against hematopoietic stem cell (HSC) senescence

This protective agent can be used as preventative or theraputic agents for alliviating related adverse effects of HSCs, such as susceptibility to infections, due to radio or chemotherapy in cancer patients. In addition, the high efficacy in HSCc tranplantation can be garanted if their senescence be diminished before or after transplantation, so, *in vitro *treatment before tranplantation and clinical intervention after transplantation can be useful to reach maximum HSC theraputic efficacy (Figure 3). 

***Mesenchymal stem cell (MSCs)***

MSCs have been distributed in many tissues including bone marrow (BM), adipose tissue, bloodstream and cord blood (85). MSCs have high self-renewal capacity and ability to differentiate into other kind of cells such as adipocytes, chondrocytes and osteoblasts depend on the organs (86). MSCs have been distributed in many adult tissues and organs such as BM, adipose tissue, umbilical cord blood and Wharton Jelly, tendon, synovial and blood circulation to control their homeostasis. MSCs in BM support hematopoietic stem cell persistence and differentiation. Also they have immunomodulatory and anti-inflammatory activities. Their proliferation rate varies between different tissues or different anatomic locations of the same tissue. BM-MSCs have less proliferative capacity than other tissues. MSC replicative senescence affects their multipotency and homing to injured or inflamed sites (87). The different sources of MSCs have shown several benefits in clinical trials. The injection of autologous BM-MSCs in patients with alcoholic cirrhosis improved their liver fibrosis and function (88). The intra-articular injection of MSCs into osteoarthritic knee improved their function and relieved its pain (89). MSCs also have been used in acute respiratory distress syndrome (90), hepatitis B virus cirrhosis(90), Crohn’s disease (91), severe diabetic foot (92), amyotrophic lateral sclerosis (93), acute myocardial infarction (94), immunomodulation after liver transplantation (95), congestive heart failure (96), chronic wounds (97), controlling blood glucose in type-2 diabetes (78) and multiple sclerosis (98). These studies have shown safety of MSC usage without any adverse effects. Before transplantation and tissue regeneration, it is necessary to culture peripheral blood MSCs in order to reach to enough number of cells for transplantation. The problem is that in higher passages telomerase activity decreases and cellular senescence increases due to p21 elevation. There is also age-dependent increasement in senescent MSCs and reduction in their proliferation capacity (99). Senescent MSCs have shown morphological changes and low self-renewal potential (100). Also elderly patients have not enough population of MSCs for efficient autologous transplantation (101). Various diseases or chronic pharmacotherapy induce senescence in MSCs. For example heparin (anticoagulant agent) and statins increased MSC senescence (102, 103). Chronic kidney disease due to premature MSC senescence showed less regeneration potential in rats (104). High glucose via Akt/mTOR signaling pathway induced MSC senescence after 14 days (105). Senescent MSCs impress their paracrine environment, immunomodulation activity, migration, differential potentials such as osteogenic and adipogenic differentiation and their therapeutic potentials negatively (106, 107). Senescent MSCs release different cytokines which can change tissue microenvironment and play role in colon cancer cells growth (108) also proliferation or migration of breast cancer cells (109). So, the reduction in MSC senescence is important for cell therapy or cancer prevention. Especially because of their wide distribution in body, there is more concern about their senescence than other type of stem cells.

*In vitro studies*

Umbilical cord blood (UCB) is one of the MSC sources. UCB-MSCs have exhibited the higher rate of cell proliferation and lower expression of P21, P53 and p16 in comparison with BM-MSCs and ASCs although they have similar levels of surface antigen expression, differentiation ability and immunosuppressive activity (110).

Wharton’s Jelly is a gelatinous substance within the umbilical cord that contains stem cells. Ten μg/ml of *Dhanwantram kashaya*, a synthetic herbal formulation, that is widely used in Ayurvedic medicine, decreased p21, cyclin D1 so senescence in MSCs of human Wharton Jelly (111). Also Preconditioning of these cells with 10 mg/ml *Tinospora cordifolia* leaf extract and 5 mg/ml *Withania somnifera* root extract down regulated p21, increased cell proliferation and delayed senescence without any toxic effects (112). 

Resveratrol (0.1,1 and 2.5 μM ) induced expression of SIRT1 and suppressed the expression of p53 and p16 thus inhibited senescence in h-UCB-MSCs. Investigattions on animals models are warranted to facilitate the clinical application of resveratrol pre-modified hUC-MSCs in treating neuro-degenerative and neural injury disorders (41). Resveratrol (0.1 mM) was optimal for inhibition of h-MSC senescence via SIRT1 in early passages. Resveratrol (0.1,1 and 2.5 mM) increased Sirt1 expression, decreased p21 and p53 thus inhibited senescence in h-UCB-MSCs. Higher concentrations of resveratrol or treatment in late passages of h-BM-MSCs induced senescence by itself by activating β-catenin. Also high concentration is corresponded to enhance Cdk2 expression. Resveratrol by activating SIRT1 inhibited phosphorylation of ERK, however, it could increase SIRT1-independent ERK activity. The activation of ERK signaling can stimulate β-catenin activity. Wnt/ β-catenin signaling through ROS production promotes the senescence of MSCs. The dose dependent protective effect of resveratrol is related to SIRT1 level in cells (40-42). H-MSCs pretreatment with 5, 10, 25, 50, 100 or 200 μM resveratrol attenuated senescence induced by H_2_O_2_. Resveratrol increased anti-oxidant activities and SIRT1 and declined pERK1/2 and p21 (113). 

Epigallocatechin-3-gallate (green tea catechin) at 50 or 100 μM concentrations exhibited protective effects against H_2_O_2_ induced senescence in h-BM-MSCs via Nrf2 activation thus decreased the acetylation of p21 and p53 (114). 

In another study, *Du-Huo-Ji-Sheng-Tang *and its active component, *Ligusticum chuanxiong, *(3 μg/ml) decreased the level of senescence after five passages in cultures of h-BM-MSCs (115). 

Ten μg/ml of *Undaria pinnatifida* ethanolic extract increased the expression of anti-oxidant enzymes such as SOD1, SOD2 and catalase and prevented the senescence of h-BM-MSCs induced by ROS in passage number of 17. It also reversed increasement in the levels of senescent proteins such as p21, p53 and p16 and declined differentiation to osteocyte and adipocyte capacity caused by cellular senescence (116).

*In vivo studies*

Twenty mg/kg/day of ginsenoside Rg1 from *Panax ginseng* increased SOD activity, reduced IL-2, IL-6, TNF-α, p16, p21, p53 and finally senescence in bone marrow stromal cells in D-galactose-induced aging rat. Aging is associated with chronic inflammation (117). DNA damages provoke NF-κB transcription factors. NF-κB transcripts inflammatory genes and induces SASP which can induce more senescent cells. IL-2, IL-6, IL-8 and TNF-α are inflammatory factors, hallmark of SASP and are secreted from senescenct cells (13).

Thirty and 100 μg/ml of *Astragalus membranaceus* polysaccharide protected mice-BM-MSCs against iron-overloaded mitochondrial ROS accumulation and senescence (118). 

**Figure 4:**
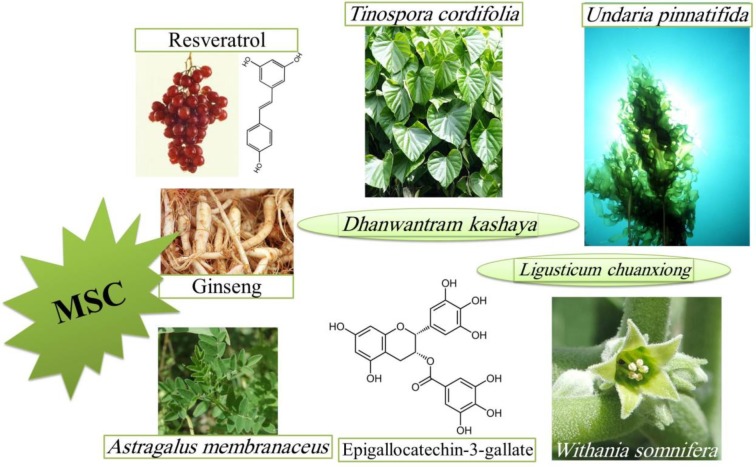
Natural agents that have shown protection against mesenchymal stem cell (MSC) senescence

Taken together, although most of these studies are *in vitro*, they are still valuable because improving the quality and the quantity of functional MSCs between harvest and transplantation plays an important role for maximum therapeutic efficacy and increases their shelf life. In cell therapy protocols, possibility for more passaging to obtain more cells without senescent phenotype is very important. Protection against the drug adverse effects can be another usage of these protective agents. Because MSCs are highly distributed in body, their senescence associated secretary factors can affect many organs and disrupt their normal functions or cause cancer formation, so inhibition of MSC senescence results in healthy body (Figure 4). 

***Adipose derived mesenchymal stem cells (ASCs)***

Adipose-derived stem cells are type of MSCs. In comparison with BM-MSCs, ASCs have some useful features such as abundant autologous sources, which are harvested easily via lipoaspiration with little pain and invasion, rapid proliferation and high proliferative capacity in cell culture. Also they are cultured easily and have shown more genetically stability (119-121). They have shown multi-lineage potentials for using in regenerative medicine and tissue engineering (121) such as osteogenesis (122), wound healing (123), slowing down Huntington disease progression (124) and urinary bladder smooth muscle engineering (124). 

Cellular senescence limits ASCs potential for therapeutic usage (125). Senescent adipose tissue secretes inflammatory cytokines (126) and it has been shown that in people with metabolic syndrome, secretion of pro-inflammatory cytokines by adipose tissue increased insulin resistance and obesity (127). Also inflammatory cytokines such as IL-6 and IL-8 can induce more cellular senescence (128).

*In vitro studies*

Five μM curcumin increased TERT expression and decreased senescence in rat ASCs (129). 

Fifty mM of resveratrol protected h-ASCs against H_2_O_2_ and D-glucose induced senescence and slowed down the rate of senescence in higher passages by attenuation of senescence-associated genes such as p21, p53, cyclin D1, IL-6 and MMP1 and increased expression of Sirt1. The paracrine effect of these cells improved insulin secretion in the rat INS-1 cells via elevation of Pim-1 expression through PI3K/Akt pathway. MMP1 is a collagenase that initiates the degradation of fibrillar collagens. Collagen provides structural support to tissues in the body. The alteration of cytoskeletal network and extracellular matrix proteins production is associated with cellular senescence. Cyclin D1 has an inhibitory effect on phosphorylated Rb which leads to cell cycle arrest (130). The improvement in insulin secretion is considerable in metabolic syndrome disease and insulin resistance managment. 

*Trans*-cinnamaldehyde (TC) is an aromatic aldehyde that exists in the bark of young *Cinnamon cassia *twigs and other cinnamon species (131). Six μM of TC decreased h-ASC senescence via Sirt1 up regulation and increased telomerase activity in ASCs so down-regulated p21 ,p53 and p16. The transfusion of these treated cells to rats showed better liver repairment than untrated cells (132). 

Five mg/ml of hydro-alcoholic guaraná (*Paullinia cupana*) extract (containing 12.240 mg/g of caffeine, 6.733 mg/g of theobromine and 4.336 mg/g of total catechins) increased SOD-1 gene expression, decreased intracellular ROS, lipoperoxidation, and protein carbonylation levels, the index of DNA damages in senescent human ASCs, also increased proliferation ability in ASC of higher passages (133).

**Figure 5:**
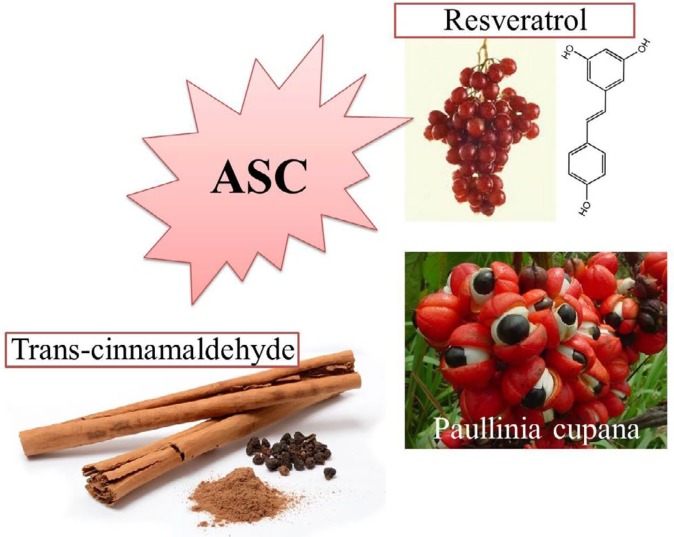
Natural agents that have shown protection against adipose-derived stem cell (ASC) senescence

The function of senescent cells has impaired and their released inflammatory molecules create harmful micro-environment for other cells which can result in more senescent cells and body organ damages. Fat tissue is highly distributed in body, so, the prevention of ASC senescence in body via supplement therapy, attenuates different diseases which are related to obesity such as insulin resistance or diabetes and improves the quality of life. The inhibition of ASC senescence in cell cultures is beneficial for more effective cell harvesting after higher passages (Figure 5).

***Keratinocyte stem cells (KSCs)***

KSCs in skin are responsible for epidermal homeostasis, starting new hair cycle, epidermal regeneration and differentiation to sebaceous glands (134-136, 29-30). The number of these cells is decreased by photoaging. Photoaging results in facial wrinkles (134-136). KSC senescence due to repeated sub-culture or chronological aging reduces epidermis reconstruction potential and makes limitation for more sub-culturing (137).

*In vitro studies*

Twenty and 100 μM of vanillin (a natural plant-derived flavor and aroma molecule) protected KSCs against UVB-induced senescence by ATM/p53/MAPK pathway. It affected MDM2 (negative regulator of the p53 tumor suppressor), so inactivated p53. Vanillin decreased the secretion of pro-inflammatory cytokines induced by UV-B, such as TNF-α, IL-1b, and IL-6 and increased anti-inflammatory cytokines such as EGF, FGF-2, and TGF-b1 (138). 

Morin is one of polyphenolic phytonutrients found in wide varieties of *Maclura pomifera*, and has been isolated from leaves of *Psidium guajava*. Morin (10, 20 and 100μM ) effectively suppressed the UVB-induced p53 specific ligasing ability of MDM2 and decreased cellular senescence in h-KSCs (138). 

**Figure 6:**
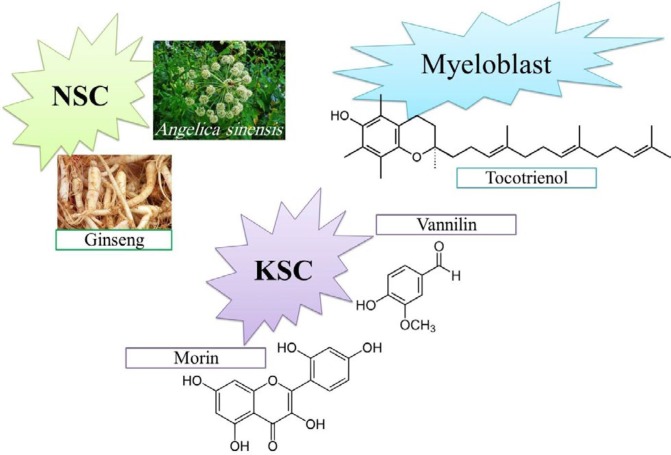
Natural agents that have shown protection against senescence of keratinocyte stem cells (KSCs), neural stem cells (NSCs) and myeloblasts

Skin is the first appearance of body aging. KSC senescence plays an important role in skin aging and induces wrinkle appearance. If they show safety for oral consumption and positive effects for other type of cells we can use these agents as protective oral supplements. Maybe they can show beneficial anti-wrinkle effects in cosmetic and topical products such as the sunscreens and skin anti-aging products in long-term usage. For using as sunscreen, their protective potential against UV-A, which is responsible for long term photoaging (139), must be evaluated. Also the role of KSC senescence in hair loss can be evaluated to discover protective agents for hair loss especially in stressful life conditions (Figure 6). 

***Other kind of adult stem cells***

*In vitro studies*

Myeloblasts are muscle stem cells and are involved in muscle regeneration which decreases by aging. Twenty-five μg/ml of tocotrienol-rich fraction diminished ROS generation and lipid peroxidation by elevating gene expression of SOD2, catalase and GPX1 and decreased senescence in myeloblasts (140). 

In the adult mammalian brain, the genesis of new neuron happens by NSCs in restricted area, hippocampus and sub-ventricular zone of the lateral ventricles (141). Neural stem cells (NSCs) are able to generate glial cells and neurons in hippocampus tissues which play an important role in learning and memory. Senescence induced by 10 mg/ml D-galactose in NSCs of mice was attenuated by 20 μg/ml ginsenoside Rg1 through elevating anti-oxidant enzymes, diminishing ROS and down-regulating Akt/mTOR signalling pathway. 

*In vivo studies*

In D-galactose aged mice, treatment with ginsenoside Rg1 increased NSCs number and improved cognitive function (142). In another study, NSCs were isolated from mice that were aged by D-galactose and were treated by Angelica polysaccharides (140 mg/kg). Then, cells were treated by 100 μg/ml Angelica polysaccharides *in vitro*. Angelica polysaccharides protected NSCs by increasing the cell proliferation; the activity of SOD and total anti-oxidant capacity, decreasing the content of malondialdehyde; the levels of IL-1b,IL-6,TNF-a and ROS; and down-regulating the expression of cellular senescence associated genes, p53 and p21, and decreased the number of senescent cells (143) (Figure 6).

**Table 1:** The summary of protective mechanisms of natural agents in senescence of stem cells

**Reference**	**Mechanisms**	**Type of study**	**Agent**
(28)	↑ Nrf2 level ↑HO-1 ↓ROS ↑Telomerase activity	*In vitro*: h-EPCs	Oleuropein and Oleacein 1.0, 2.0 and 5.0 μM
(29)	↑ Nrf2 level ↑HO-1 ↓ROS ↑Telomerase activity	*In vitro*: h-EPCs	Anthocyans-rich *A. melanocarpa *extract 1–25μg/ml
(30)	↑Telomerase activity	*In vitro*: h-EPCs	Ginsenoside Rg1 5 μM
(31)	Not studied	*In vitro*: h-EPCs	Sun Ginseng 200 μg/ml
(32)	↑Phosphorylation of Akt ↑ Telomerase Activity	*In vitro*: h-EPCs	Ginkgo biloba extract 25 mg/L
(33)	↑Phosphorylation of Akt ↑ Telomerase activity	*In vitro*: h-EPCs	Puerarin 1. 0 mM
(36)	↑hTERT protein ↑Telomerase activity ↑ PPARγ/ HO1 ↓ROS	*In vitro*: h-EPCs	Resveratrol 0. 110 μM
(37)	↑Phosphorylation of Akt ↑Telomerase activity	*In vitro*: h-EPCs	Resveratrol 10, 25 or 50 mM
(38)	↑ PI3-K/Akt ↑ h-TERT expression ↑ Telomerase activity	*In vitro*: h-EPCs	Resveratrol 1, 10, and 50 µM
(44)	↑ Akt/h-TERT ↑Telomerase activity ↓ NADPH oxidase	*In vitro*: BM- EPCs of rat	Polysaccharides of *A. sinensis* 100 μg/ml an 20 μg/ml
(45)	↑Nrf-2 Translocation ↑GPx-1 expression ↑ SOD2 expression ↑HO-1 expression ↓ ROS generation	*In vitro*: h-EPCs	*T. Sativum *grain And Bean lysate 0. 35 and 0. 7 mg/ml
(49)	↑Nrf-2 Translocation ↑HO-1 protein ↓Oxidative stress	*In vitro*: h-EPCs	Red Yeast Rice 50 μg/ml
(50)	↑Telomerase activity	*In vitro*: h-EPCs	Silymarin 12. 5–50 mg/ml
(52)	↑SMP30 ↑Akt phosphorylation ↑ERK phosphorylation ↓p21	*In vitro*: h-ECFC	Fucoidan (0, 0. 1, 1, 10, and 30 μg/ml)
(57)	Not studied	*In vivo*: EPCs of type1 diabetic mice	Curcumin 1000 mg/kg/day
(58)	↑Pi3K/Akt/enos signaling	Clinical: h-EPCs	Red wine 250 ml/day for 3weeks
(59)	↑eNOS/NO	Clinical and *in vitro*: h-EPCs	Red wine 100 ml/day for 3 weeks *In vitro*: Red wine 1% or Resveratrol 50 μM
(69)	↑TERT expression ↑Telomerase activity	*In vitro*: HSCs of umbilical cord blood	Tianshengyuan-1 31. 2 μg/ml and 62. 5 μg/ml
(70)	↓p16 mRNA and protein ↑ Telomerase activity ↑ Telomere	*In vitro*: HSCs	Ginsenoside Rg1 10 μM
(71)	↑Sirt6 ↓ NF-KB	*In vitro*: HSCs of mice	Ginsenoside Rg1 10 μM
(74)	↓P16 ↓Rb ↑ CDK2 ↑ CDK4 ↑cyclin E ↑Telomerase activities	*In-vitvo*: Sca1+ hematopoietic cells *In vivo*: Mice	Ginsenoside Rg1 10 μM
(77)	↓p16-Rb ↓p19-p53-p21 ↓Cyclin D1	*In vivo*: HSCs of irritated mice	Ginsenoside Rg1 20mg/kg
(77)	↑SOD	*In vivo*: HSCs of irritated mice	Ginsenoside Rg1 20mg/kg. Day for 7 days
(73)	↑Sirt1 ↓NF-KB	*In vivo*: HSCs of D-galactose aging model rat	Ginsenoside Rg1
(78)	↑SOD activity ↓ DNA damages ↓ P16 ↓ P21	*In vivo*: HSCs of irritated mice	Ginsenoside Rg1 20 mg·kg-1·d-1
(79)	↑ nrf-2 ↓ROS ↓ p16	*In vivo*: HSCs of irritated mice	Theaflavin 50 mg/kg
(80)	↓ROS level ↓ p21, p53 and p16.	*In vivo*: HSCs of mice	*S. grosuenorii* 3g/day
(76)	↓NOX4 ↑Sirt1 ↑ SOD1 ↑ GPx1 ↓ROS ↓p16	*In vivo*: HSCs of mice	Resveratrol 20 mg/kg
(81)	Not stablished	*In vivo*: HSCs of mice	6g/kg Astragalus and 3g/kg Angelica
(68)	↑Telomerase activity ↓p53	*In vivo*: HSCs of irritated mice	sinensis Polysaccharides 200mg/kg
(82)	↑Antioxidant capacity ↓DNA damages ↓P16-RB ↓ P19-P21 ↓ Wnt/beta-catenin signaling	*In vivo*: HSCs of Mice D-galactose induced aging	*A. sinensis* polysaccharides 200mg/kg
(144)	↓ mTOR ↑Bmi-1 ↓p16	*Ex vivo:* BM-HSCs of mice	Rapamycin 20 ng/ml
(145)	NOX4↓ ↑SOD1, SOD2, CAT, GPX1	*In vivo*: HSCs of mice	Metformin 250 mg/kg/day
(111)	↓P21	*In vitro*: MSCs of human Wharton jelly	*Dhanwantram kashaya* 10 μg/ml
(112)	↓P21	*In vitro*: MSCs of human Wharton jelly	*T. cordifolia *leaf 10 mg/ml *W. somnifera *root extracts 5 mg/ml
(41)	↑Sirt1 ↓p21 and p53	*In vitro:* h-UCB-MSCs	Resveratrol 0. 1, 1 and 2. 5 mM
(40, 42)	↑SIRT1 ↓p21 and p53	*In vitro*: h-MSCs h-BM-MSCs	Resveratrol 0. 1mM
(113)	↑ SIRT1 ↑Antioxidant activity ↓pERK1/2 ↓p21	*In vitro*: h-MSCs	Resveratrol 0, 5, 10, 25, 50, 100 or 200 μM
(114)	Nrf2 activation ↓ p21 and p53	*In vitro*: h-BM-MSCs	Epigallocatechin-3-gallate 50 or 100 μM
(115)	Not studied	*In vitro*: h-BM-MSCs	Du-Huo-Ji-Sheng-Tang *Ligusticum chuanxiong *(3μg/ml)
(116)	↑SOD and catalase ↓ROS ↓P21,p53,p16	*In vitro*: h-BM-MSCs	*U. pinnatifida* ethanolic extract 10 μg/ml
(117)	↑SOD ↓ p16, p21, p53	*In vivo*: MSCs of Rat	Ginsenoside Rg1 20 mg/kg · d
(118)	↓ROS	*In vitro*: BM-MSCs of mice	*membranaceus *polysaccharide 30 and 100 μg/ml
(129)	↑ TERT expression	*In vitro:* rat-ASCs	Curcumin 5μM
(130)	↑sirt1 ↓p21 ↓p53 ↓cyclin D1	*In vitro:* h-ASCs	Resveratrol 50mM
(132)	↑Sirt1 ↑Telomerase activity ↓p21 ,p53 and p16	*In vitro:* h-ASCs	*Trans*-cinnamaldehyde 6 μM
(133)	↑SOD-1 ↓ ROS ↓ DNA damages	*In vitro:* h-ASCs	Hydro-alcoholic guaraná (*P. cupana*) extract 5 mg/ml
(138)	↓ATM/p53/MAPK	*In vitro*: KSCs of human fore skin	Vannilin 20, 100 μM
(138)	↑MDM2 ↓p53	*In vitro*: KSCs of human fore skin	Morin 10, 20, 100 μM
(140)	↑ SOD2, GPX1 ↓ROS	*In vitro*: Myeloblasts	Tocotrienol-rich fraction 25 𝜇g/ml
(142)	↑anti-oxidant enzymes ↓ROS ↓ Akt/mTOR signalling	*In vitro*: NSCs of mice	ginsenoside Rg1 20 𝜇g/ml
(143)	↑SOD ↑total antioxidant capacity ↓malondialdehyde ↓IL-1b,IL-6,TNF-a ↓ROS ↓p53, p21	*In vitro*: NSCs of mice	Angelica polysaccharides (140 mg/kg *in vivo *then 100 𝜇g/ml *in vitro *)

**Figure 7:**
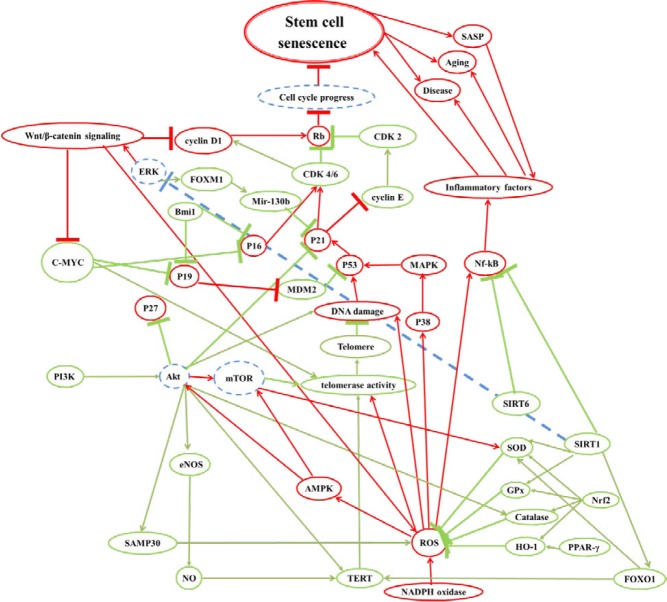
The schematic of proteins which are involved in stem cell senescence

## Discussion

Adult stem cells are distributed in whole body and play critical roles in tissue regeneration and health maintaining. We should consider stem cell senescence in different conditions such as aging, diseases, adverse drug effects and *in vitro *expansion for cell therapy. If we recognize involved pathways in their senescence, then we can counteract with them (Figure 7). As reviewed in this paper most protective agents finally increased telomerase activity or decreased oxidative damages via various molecular mechanisms which inhibited cellular senescence.

In cell culture or body, we cannot separate young cells from senescent cells, which secrete harmful cytokines and affect whole body, so, we should prevent their formation. Senescence inhibition in body results in health and longevity. There are many natural agents, which inhibit senescence through different mechanisms. Among them ginsenoside Rg1 and resveratrol were the most studied agents. Some of agents have shown desirable effects in different kinds of stem cells. Although, most of these studies were *in vitro, *they are still valuable because they can be considered in cell therapy for increasing stem cell shelf life and function, so, bring more successes in the clinic. Moreover, *in vitro *studies are the first step toward clinical studies. 

## Conclusion

Due to safe and long history usage of plants in clinic and experiments, these agents can be used as supplement for preventing stem cell senescence and their related complication *in vitro* and *in vivo*. Resveratrol and ginseng can be the first choice for this aim due to their protective effects in various kinds of stem cells.

## Conflicts of Interest

The authors declare that there are no conflicts of interest.
